# Book Review: “Cognitive neuroscience of language”

**DOI:** 10.3389/fpsyg.2015.00553

**Published:** 2015-05-01

**Authors:** Patrick C. Trettenbrein

**Affiliations:** ^1^Language Development & Cognitive Science Unit, Department of English Studies, University of GrazGraz, Austria; ^2^Department of Linguistics, University of GrazGraz, Austria

**Keywords:** linguistics, cognitive neuroscience, neurolinguistics, aphasiology, language and the brain, neuroimaging, textbook

The 19^th^ century has brought to linguistics “Broca's problem” (Boeckx, 2009), the question of how knowledge of language is implemented in the brain. Since then neuroscientific work on language was mostly driven by aphasiology, a state of affairs that has changed drastically with the advent of cognitive neuroscience toward the end of the 1970s and 1980s (Gazzaniga, 2015) and the subsequent further technical advancement and establishment of neuroimaging. With the publication of the textbook *Cognitive Neuroscience of Language* David Kemmerer aims at and succeeds in closing a long-standing gap in the available neurolinguistics literature, taking into account that cognitive neuroscience has come of age and progressed considerably in addressing Broca's problem in the relatively short timespan since its establishment.

Already a brief glimpse at the table of contents reveals that Kemmerer's book does not just cover “language” in the narrow sense of many linguists (Hauser et al., 2002). Also, the book is intended to be a textbook for graduate and advanced undergraduate students which is why it follows an according chapter layout. Consequently, the first chapters offer a general introduction to the workings of the human brain, as well as an overview over the different methods for mapping it that are being used in the field (e.g., positron emission tomography, (functional) magnetic resonance imaging, and magnetoencephalography). This is followed by a thorough discussion of what was and still is being learned from the investigation of aphasic syndromes, before Kemmerer moves on to attend to a general discussion of speech production and perception which also includes a separate chapter on prosody covering common linguistic as well as emotional prosody. Demonstrating the book's scope, the author's course of action continues with chapters dedicated to what he calls “other modalities,” whereas he limits this not only to sign language but also addresses reading and writing.

Following these rather holistic preceding chapters, the book continues with cutting-edge considerations of the neural underpinnings and correlates of the linguist's familiar and beloved semantics, morphology, and syntax. It is especially noteworthy that Kemmerer is very much aware of the current state of linguistic research in regard to linguistic diversity in all these subfields and incorporates them into his descriptions. Then, step by step, the author moves along from discussing the production and processing of individual sentences to the discourse level. Prudently, the author's treatment of these classical areas of linguistic investigation such as, for example, syntax is largely or even fully “agnostic” about linguistic theory and focuses solely on the neuropsychological studies and evidence available. Many a reader with a classical background in linguistics would possibly have hoped for a more thorough treatment of linguistic theory, but as a unification of linguistic theory and cognitive neuroscience is still pending (the so-called “granularity mismatch problem;” Poeppel and Embick, 2005/2013) Kemmerer's “agnostic” stance seems very much appropriate and indicated.

As should be expected from a textbook, throughout the author has taken great care to ensure accessibility to the non-expert by extensively explaining all technical terms and concepts whenever they are employed for the first time. Color-coded info boxes provide concise definitions of utilized terminology, whereas regularly reappearing larger boxes are devoted to providing brief summaries of subject matters directly related to a chapter's contents. In addition, at the end of every chapter Kemmerer has included a summary of key points and up-to-date suggestions for further reading. Most pleasantly, the book is consistently equipped with a myriad of color pictures that mainly depict the respectively discussed findings from neuroimaging, thus making it easy to follow the author's train of thought and line of argument by looking at the evidence oneself. Generally speaking, the range of the evidence on which Kemmerer draws in this book and his knowledge of both, (cognitive) neuroscience and linguistics, is impressive and makes for substantiated and scientifically sound reading.

Ordinarily, a thorough book review also ought to address the identified flaws of a publication, however, this is much more easily said than done in the case of Kemmerer's book as there are no obvious shortcomings. In lack thereof, from a linguist's perspective, one might criticize the choice of title as Kemmerer has put together such an extensive collection of current research in the field that is by no means limited only to language (in many linguists' narrow sense). In point of fact, the multitude of studies portrayed and discussed in *Cognitive Neuroscience of Language* reminds us that “language” (unfortunately) does not exist in a neural void but supervenes and interferes with what appears to be the majority of cognitive systems. Hence, the title “Cognitive Neuroscience of Language *and Communication*” would probably have been more appropriate to signal the book's magnitude to the inclined reader.

To conclude, Kemmerer's book is a genuine tour de force, capturing the current state of research that is addressing Broca's problem in the widest sense. While definite answers are still missing, this book provides researchers with a comprehensive and state-of-the-art compilation of cognitive neuroscience research on language and communication. Though Kemmerer's intention was to write a textbook for graduate students what he has actually achieved goes far beyond this primal ambition. As a matter of fact, Kemmerer's book makes not just for a useful textbook for teaching graduate students, but might also be employed as an up-to-date work of reference for the aspiring early-career researcher. What is more, newcomers and interested outsiders are well-advised to refer to *Cognitive Neuroscience of Language* if they want to gain an overview of the current state of research in the field.

## Conflict of interest statement

The authors declare that the research was conducted in the absence of any commercial or financial relationships that could be construed as a potential conflict of interest.
